# Respiratory tract infections and gut microbiome modifications: A systematic review

**DOI:** 10.1371/journal.pone.0262057

**Published:** 2022-01-13

**Authors:** Claire A. Woodall, Luke J. McGeoch, Alastair D. Hay, Ashley Hammond

**Affiliations:** 1 Centre for Academic Primary Care, Bristol Medical School, Population Health Sciences, University of Bristol, Bristol, United Kingdom; 2 Nuffield Department of Population Health, University of Oxford, Oxford, United Kingdom; Imperial College London, UNITED KINGDOM

## Abstract

Respiratory tract infections (RTIs) are extremely common and can cause gastrointestinal tract symptoms and changes to the gut microbiota, yet these effects are poorly understood. We conducted a systematic review to evaluate the reported evidence of gut microbiome alterations in patients with a RTI compared to healthy controls (PROSPERO: CRD42019138853). We systematically searched Medline, Embase, Web of Science, Cochrane and the Clinical Trial Database for studies published between January 2015 and June 2021. Studies were eligible for inclusion if they were human cohorts describing the gut microbiome in patients with an RTI compared to healthy controls and the infection was caused by a viral or bacterial pathogen. Dual data screening and extraction with narrative synthesis was performed. We identified 1,593 articles and assessed 11 full texts for inclusion. Included studies (some nested) reported gut microbiome changes in the context of Severe Acute Respiratory Syndrome Coronavirus 2 (SARS-CoV-2) (n = 5), influenza (H1N1 and H7N9) (n = 2), Tuberculosis (TB) (n = 4), Community-Acquired Pneumonia CAP (n = 2) and recurrent RTIs (rRTI) (n = 1) infections. We found studies of patients with an RTI compared to controls reported a decrease in gut microbiome diversity (Shannon) of 1.45 units (95% CI, 0.15–2.50 [*p*, <0.0001]) and a lower abundance of taxa (p, 0.0086). Meta-analysis of the Shannon value showed considerable heterogeneity between studies (I^2^, 94.42). Unbiased analysis displayed as a funnel plot revealed a depletion of Lachnospiraceae, Ruminococcaceae and *Ruminococcus* and enrichment of *Enterococcus*. There was an important absence in the lack of cohort studies reporting gut microbiome changes and high heterogeneity between studies may be explained by variations in microbiome methods and confounder effects. Further human cohort studies are needed to understand RTI-induced gut microbiome changes to better understand interplay between microbes and respiratory health.

## Introduction

Respiratory tract infections (RTIs) are ubiquitous in society and place a high burden on the healthcare system. Prior to the COVID-19 pandemic, mean annual healthcare spending on acute RTIs was £86 million [1]. The COVID-19 pandemic has resulted in enormous health and societal implications, and is currently attributed to 3.7 million deaths worldwide [2]. RTIs are caused by bacterial, viral and fungal pathogens and symptoms are not limited to the respiratory tract, with gastrointestinal symptoms such as diarrhoea and cramps being common sequelae [3].

Microbial communities are associated with many human body niches, including the gut, skin, lungs, and mucosal surface [4–6]. The gut is the most densely and diversely colonised organ, with a bacterial to host cell ratio of 1:1 [7]. A healthy adult gut microbiota consists mainly of the phyla Firmicutes including genera, *Roseburia*, *Enterococcus* and *Faecalibacterium* and Bacteroidetes including predominant genera such as *Bacteroides* and *Prevotella* [8, 9]. Gut bacteria synthesise vitamins, aid in nutrient metabolism and mediate immunomodulation [10]. However, the diversity and abundance of healthy gut microbiota can be disrupted by factors, leading to gut dysbiosis which can involve pathobiont proliferation and depletion of commensal bacteria [11]. These gut microbiota alterations have been associated with several diseases including diabetes, asthma, colorectal cancer and Parkinson’s disease [12–15].

A plethora of evidence supports systemic microbial communication between microbiota of the respiratory tract and gastrointestinal tract through the circulatory and immune system [16]. Known as the ‘*gut-lung axis*’, this bi-directional system is complex and involved in both health, disease and secondary disease outcomes [17]. For example, gut microbiome derived short chain fatty acids (SCFAs) such as acetate, propionate and butyrate from dietary fermentation, in combination with host-derived cytokines and chemokines, travel via the bloodstream and lymphatic system and are directly associated with a protective response [5]. Infections can also influence this. For example, influenza virus infection of the respiratory tract results in systemic immune signals which modulate gut microbiota, leading to outgrowths of *Escherichia coli* even in the absence of detectable influenza virus in the gut [18]. Conversely, acetate-producing gut bacteria can protect against respiratory syncytial virus (RSV) infection through an interferon response [19]. It is becoming increasingly evident that infectious RTIs can directly affect gut microbiota however the details are not yet fully understood.

This systematic review aims to evaluate current reported evidence for the impact of a RTI on microbiome abundance and diversity in patients with a confirmed or suspected RTI compared to healthy controls.

## Materials and methods

We conducted and reported this systematic review according to the Preferred Reporting Items for Systematic Reviews and Meta-Analysis statement and registered the protocol prospectively with PROSPERO (CRD42019138853).

### Search strategy and eligibility criteria

In June 2021 we performed a systematic search of MEDLINE (OvidSP), EMBASE (OvidSP) Web of Science (WoS) and Clinical trial databases including the World Health Organisation (WHO) International Clinical Trial Database, United Kingdom (UK) Government clinical trials register and the Cochrane Central Register of Controlled Trials (CENTRAL) to identify studies in humans that compared the gut microbiome profiles of RTI patients to those of healthy controls. The search covered the years 2015–2021 as microbiome data have been most consistently reported during this period. The search strategy used a combination of Medical Subject Heading (MeSH) terms and words for types of RTIs and microbiological data. The search was done twice. In the first instance with simple words and terms whereas the second search included target words to obtain recent Covid-19 related publications. The full search strategy can be seen in S1a Table and key words in S1b Table.

The full inclusion/exclusion criteria are described in S2 Table. Briefly, studies eligible for inclusion were peer-reviewed original research studies reporting gut microbiome data from patients aged over 3 years old with a suspected or proven clinical diagnosis of, or symptoms in keeping with, an RTI compared to healthy controls. Participant recruitment was from primary care, secondary care and community settings in any country. Studies were excluded if they were lacking gut microbiome data, non-human, neonates, intervention studies investigating the impact of probiotics, anxiety, diet, body mass index, drug or antibiotic usage, environmental factors such as smoking or if participants had a history of respiratory disease. A further 5 studies were excluded based on medication, antiviral or antibiotic usage in the majority of patients and did not consider these factors as a confounder, also one study did not provide a baseline or comparison with healthy control participants [20–25].

### Study selection

PICOS criteria were used to guide one reviewer (CAW) to screen all titles and abstracts for eligibility, and a second reviewer (AH) to perform a 10% double-screen. Full-text copies of included articles were independently reviewed. Dual screening was performed for all records by two authors (AH and LM) and eligibility disagreements were resolved by discussion.

### Data extraction and quality assessment

Gut microbiome data from all studies were extracted into a purpose-built Microsoft Excel (vs 16.48) spreadsheet, summarised in S3 Table (S1 Appendix). Collected data contains descriptive variables including country of recruitment, study setting, study design, DNA extraction methods, sequence platform, bioinformatics pipelines, gut bacteria identified, number of RTI patients, number of healthy controls, age of patient and RTI causative microbe.

To assess study quality and risk of bias, the Critical Appraisal Skills Programme (CASP) checklist, including 12 questions for cohort and 10 questions for case-control studies was applied to each study. We produced a quality assessment chart based on a traffic light system of ‘good’, ‘adequate’ or ‘poor’, as recommended by Cochrane [26].

### Data synthesis and analysis

All taxa including phyla, family, genus and species of gut bacteria in RTI patients which were also reported in the control participants were recorded. Those bacteria reported as family, genus or species were allocated into the corresponding phylum. The relative abundance of each phylum was calculated as a percentage. The variation in relative abundance of each gut bacterium in RTI patients, compared to healthy controls, was reported as ‘increase’, ‘decrease’ or ‘no change’.

In line with Cochrane recommendations, and where the data allowed, meta-analysis was conducted. To assess microbiota heterogeneity the alpha diversity data was compared between studies using the most commonly reported diversity value, the Shannon Diversity Index (DI), a measure of abundance and evenness of taxa. Other alpha diversity measures including the Operational Taxonomic Units (OTUs, the richness count of different species) and Chao1 (estimate of diversity from abundance data) were collated. Where there was an insufficient number of studies, or non-homogeneous data between studies, data was described descriptively.

For specific gut bacteria, a funnel plot was used to compare the proportion of studies that reported either an increase or decrease out of the total number of studies in which they were reported (S3 Table). The proportion data was processed in in RStudio (version 1.3.1073) using the funnelR script which is based on a binomial Poisson distribution score2 and significance levels set at 50%, 80% and 95% confidence limits (Cl) [27].

## Results

**Fig 1:**
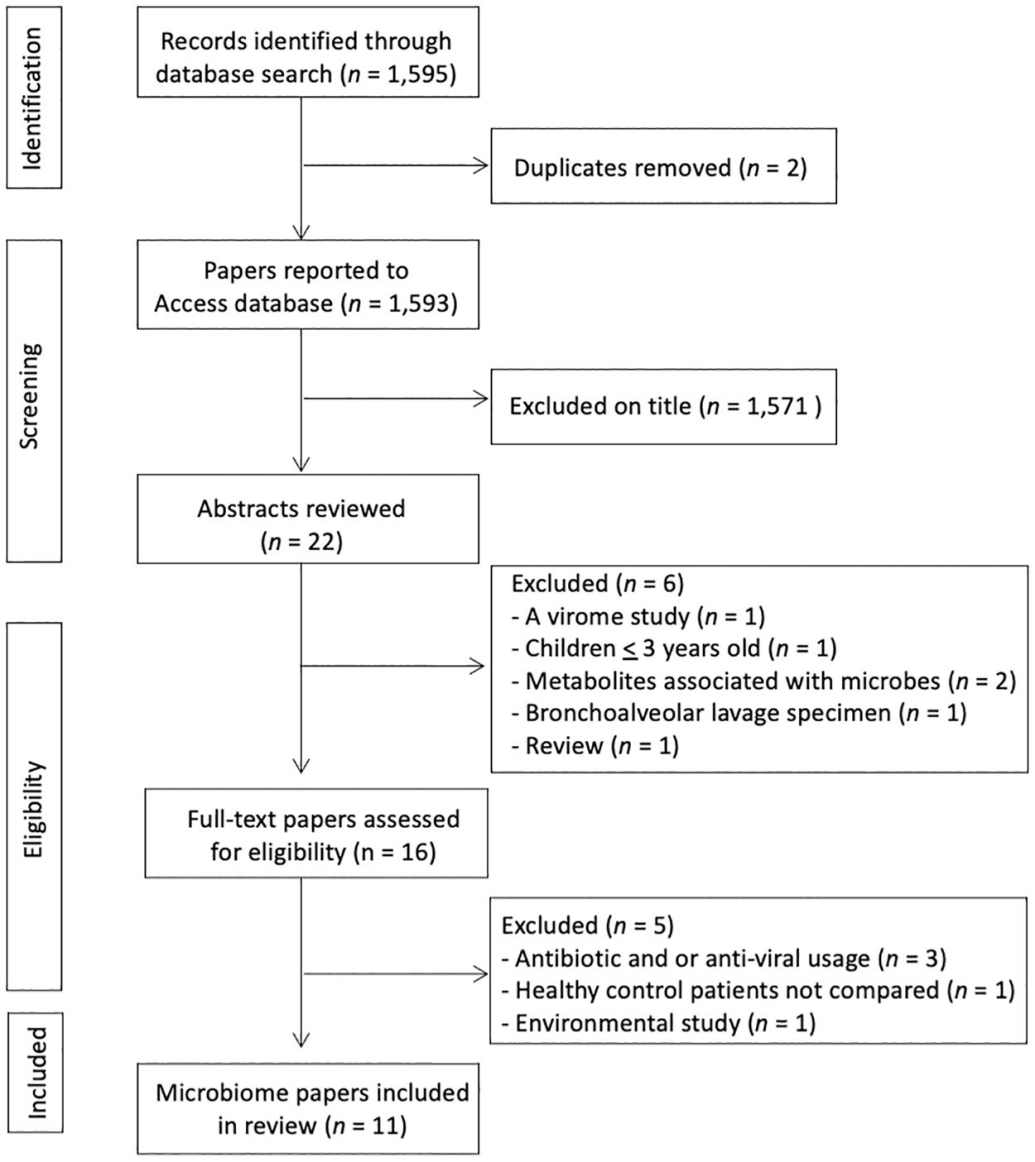
Data search and extraction (PRISMA flow chart).

The search strategy identified 1,595 articles, of which 2 were duplicates. A preferred reporting items for systematic reviews and meta-analyses (PRISMA) data search and extraction was completed showing exclusion decisions (Fig 1 and S1 Appendix). Of the remaining 1,593 articles, 1,571 were screened by title and abstract from which 22 articles were identified as meeting the inclusion criteria. Following an abstract review, a further 6 papers were excluded based on the PICOS exclusion criteria. A total of 16 articles were reviewed in full and 6 articles were excluded based on the PICOS criteria, leaving 11 papers eligible for inclusion in this review. In addition, a search of three clinical trial databases resulted in a total number of 66 studies, with 1 article cross-matched to the main search.

### Risk of bias assessment

Study quality and risk of bias for all included studies were scored against cohort and case-control studies (S1 Fig). All included studies were of suitable quality and demonstrated unrestricted bias.

### Main study characteristics

The characteristics of included studies are shown in Table 1.

**Table 1:** Main characteristics of included studies.

First author (year)	Study Title	Country	RTI Pathogen	Age category (age yrs)^a^	Participants		Microbiome approach, pipeline and database^c^	Diversity measures and analysis^c^
					RTI patients^b^	Healthy controls		
Ren Z., *et al*., (2021)	Alterations in the human oral and gut microbiomes and lipidomics in Covid-19.	China	SARS-CoV-2	Adult (48 ± 10.24)	24	48	16S rRNA (V3-V4) UPARSE RDP classifier	Shannon DI, PCoA, Wilcoxon rank-sum test, Fisher’s test, POD, ROC and AUC analysis.
Newsome *et al*., (2021)	The gut microbiome of COVID-19 recovered patients returns to uninfected status in a minority-dominated United States cohort	USA	SARS-CoV-2	Adult (62)	50	34	16S rRNA (V1-V3) DADA2 SILVA	Shannon DI, PCoA, PERMANOVA, Log2FC
Yeoh *et al*., (2021)	Gut microbiota composition reflects disease severity and dysfunctional immune responses in patients with Covid-19	China	SARS-CoV-2	Adult (36 ± 18.7)	53	78	Metagenomics MetaPhlAn2	PCA PERMANOVA Procrustes LEfSe MaAsLin
Gu *et al*., (2020)	Alterations of the Gut Microbiota in Patients with Covid-19 or H1N1 Influenza.	China	SARS-CoV-2	Adult (55)	30	30	16S rRNA (V3-V4) UPARSE SILVA	Shannon DI Chao1 PcoA, Bray-Curtis
			Influenza (H1N1)	Adult (48.5)	24			
Zuo *et al*., (2020)	Alterations in Gut Microbiota of Patients With Covid-19 During Time of Hospitalization.	China	SARS-CoV-2	Adult (55)	7	15	Metagenomics MetaPhlAn2	Bray-Curtis MaAsLin Spearman
			CAP		6			
Ren *et al*., (2020)	The distribution characteristics of intestinal microbiota in children with community-acquired pneumonia under five years of age.	Inner Mongolia	CAP	Children (4–5)	11	10	16S rRNA (V4) QIIME2 Greengenes	Shannon DI Simpson DI Chao1 ANOSIM
Li L., *et al*., (2019)	Intestinal microbiota dysbiosis in children with recurrent respiratory tract infections.	China	rRTI	Children (>5)	26	23	16S rRNA (V3-V4) UPARSE mothur USEARCH	Shannon DI Simpson DI Chao1 ACE NMDS, Bray-Curtis
Hu *et al*., (2019)	The Gut Microbiome Signatures Discriminate Healthy From Pulmonary Tuberculosis Patients.	China	TB	Adults (28 + 2.2)	30	31	Metagenomics MetaPhlAn2	Shannon DI NMDS MOCAT2, MetaPhlAn2, HUMAnN2 MetaCyc
Li W., *et al*., (2019)	Characterization of gut microbiota in children with pulmonary tuberculosis.	China	TB	Children (6 + 0.2–15.5)	18	18	16S rRNA (V3-V4) QIIME2 Greengenes	Shannon DI Simpson DI Chao1 ACE
Luo *et al*., (2017)	Alternation of gut microbiota in patients with pulmonary tuberculosis.	China	New TB	Adult (35–47)	19	20	16S rRNA (V4) UPARSE & QIIME Greengenes	Shannon DI Simpson DI Chao1 ACE PcoA, UniFrac
			Recurrent TB		18			
Qin *et al*., (2015)	Influence of H7N9 virus infection and associated treatment on human gut microbiota.	China	Influenza (H7N9)	Adult (57)	9	31	Metagenomics Casava Strainseeker	Shannon DI PCoA eggNOG KEGG

^a^Cohorts reported as either adult or children. Mean age (unless otherwise stated).

^b^RTI patients gut microbiome data were considered only if patients were reported as not taking antibiotics.

^c^Shannon Diversity Index (DI), Simpson’s Diversity Index (DI), Chao1, Abundance-based Coverage Estimator (ACE), Bray-Curtis, Log2FC (fold change), UniFrac, analysis of similarity test (ANOSIM), Metagenomic Phylogenetic Analysis (MetaPhlAn2), UPARSE, mothur, SILVA, Greengenes, Strainseeker, USEARCH (rRNA sequence databases), Quantitative Insights Into Microbial Ecology (QIIME2), DADA2, Principal Component Analysis (PCA) and nonmetric multidimensional scaling (NMDS), permutational multivariate analysis of variance (PERMANOVA) and Procrustes, linear discriminant analysis effect size (LEfSe), multivariate analysis by linear models (MaAsLin), Metabolic Pathway Databases (MetaCyc, eggNOG, Kyoto Encyclopaedia of Genes and Genomes [KEGG], Ribosomal database project (RDP), Probability of Disease (POD), Receiver operating characteristic (ROC), area under the ROC curve (AUC). Other abbreviations: Respiratory Tract Infection (RTI); Pathogens, SARS-CoV-2, Tuberculosis (TB), Community-Acquired Pneumonia (CAP), Recurrent-RTI (rRTI), Influenza A virus subtype H1N1 (H1N1), Influenza A virus subtype H7N9 (H7N9).

Ten studies were conducted in China and one in the United States of America. One study recruited participants from the community and 10 studies recruited hospital patients (either admitted or recently discharged). One study recruited two child participant groups (0–3 years and 4–5 years of age) and as the gut microbiome in young children (<3 years) is known to be immature [28], we only considered the gut microbiome of the 4–5 years age group for inclusion in this review. Antibiotics, antivirals and over-the-counter medications are known to perturb gut microbiota [29]. Studies which appeared relevant yet reported usage of these medications in patients with an RTI were excluded (S1 Appendix). Three included articles reported patients with an RTI who were taking medications compared to healthy controls and adjusted gut microbiome analysis [23, 30, 31].

All studies reported that stool samples were collected from participants as a proxy for gut microbiota analysis. Stool samples were collected into a sterile ‘stool tube’ that was immediately frozen at -80°C following sample receipt. One study did not report stool storage or transport conditions [30]. The extraction methodology for bacterial genomic DNA (gDNA) was variable. The following commercial kits were used: DNeasy PowerSoil and QIAamp DNA Stool Mini Kit (QIAGEN), PSP^®^ Spin Stool DNA Plus Kit (STRATEC Molecular), E.Z.N.A.^®^Stool DNA Kit and OMEGA stool DNA mini kit (OMEGA), Maxwell RSC PureFood GMO and Authentication Kit (PROMEGA).

The two main approaches for microbiome analysis are metagenome sequencing and amplicon sequencing targeting the 16S rRNA gene (16S). Metagenome libraries were created in four studies, whereas the rest of the studies carried out 16S profiling. Of these, two studies amplified the V4 region, four studies amplified the V3-V4 region, and one study amplified the V1-3 region. A range of bioinformatic tools are available for microbiome analysis. Two studies applied Quantitative Insights Into Microbial Ecology (QIIME2) [32], four used UPARSE [33], one used mothur [34] and one used DADA2 [35]. A range of different 16S and Archaea databases were used to assign the bacterial names from amplicon sequence variants (ASVs) including Greengenes [36], SILVA [37], Strainseeker [38], USEARCH [39], Ribosomal database project (RDP) [40] and Consensus Assessment of Sequence And VAriation (CASAVA) [41]. In three metagenome studies the Metagenomic Phylogenetic Analysis (MetaPhlAn2) [42] package was used to determine taxa from whole genome libraries. Two studies performed metabolic pathway analysis tools using MetaCyc [43], eggNOG-Mapper [44] and Kyoto Encyclopaedia of Genes and Genomes (KEGG) [45].

### Microbiome characteristics

The total number of reported taxa of gut bacteria in RTI patients was 115, including 8 phyla, 17 families, 55 genera and 35 species, Table 2 (and S3 Table).

**Table 2:** Gut microbiome in patients with a respiratory tract infection compared to healthy participants.

First author and year	RTI pathogen	Variation abundance	Gut taxa reported in RTI patients compared to healthy participants			
			Phylum	Family	Genus	Species
Ren Z., *et al*., 2021	SARS-CoV-2	Increase			*Enterococcus*, *Streptococcus*	
		Decrease		Ruminococcaceae *Lachnospiraceae*	*Bacteroides*, *Bifidobacterium*, *Blautia*, *Faecalibacterium*, *Pseudobutyrivibrio*	
		No change			*Akkermansia*	
Newsome *et al*., 2021	SARS-CoV-2				*Corynebacterium*, *Campylobacter*, *Finegoldia*	
Yeoh *et al*., 2021	SARS-CoV-2	Increase	Bacteroidetes			*Akkermansia muciniphila* *Bacteroides dorei* *Bacteroides ovatus* *Bacteroides vulgatus* *Bacteroides caccce* *Ruminococcus gnavus* *Ruminococcus torques*
		Decrease	Actinobacteria			*Bifidobacterium adolescentis* *Bifidobacterium pseudocatenulatum* *Collinsella aerofaciens* *Coprococcus comes* *Dorea longicatena* *Eubacterium rectale* *Faecalibacterium prausnitzii* *Ruminococcus bromii* *Ruminococcus obeum* *Subdoligranulum sp*.
Gu *et al*., 2020	SARS-CoV-2	Increase		Actinomycetaceae Micrococcaceae Streptococcaceae	*Streptococcus*	
		Decrease	Actinobacteria Firmicutes	Bifidobacteriaceae Coriobacteriaceae Erysipelotrichaceae Lachnospiraceae Peptostreptococcaceae Ruminococcaceae	*Agathobacter* *Anaerostipes* *Bifidobacterium* *Blautia* *Collinsella* *Dorea* *Erysipelotoclostrium* *Faecalibacterium* *Fusicatenibacter* *Intestinibacter* *Romboutsia* *Ruminococcus*	*Clostridium stricto 1* *Eubacterium hallii* *Ruminococcus torques*
		No change			*Rothia*	
Gu *et al*., 2020	Influenza H1N1	Increase		Actinomycetaceae Micrococcaceae		
		Decrease	Actinobacteria Firmicutes	Bifidobacteriaceae Coriobacteriaceae Erysipelotrichaceae Lachnospiraceae Peptostreptococcaceae Ruminococcaceae	*Agathobacter* *Anaerostipes* *Bifidobacterium* *Blautia* *Collinsella* *Dorea* *Erysipelotoclostrium* *Faecalibacterium* *Fusicatenibacter* *Intestinibacter* *Romboutsia* *Ruminococcus* *Streptococcus* *Rothia*	*Clostridium stricto 1* *Eubacterium hallii* *Ruminococcus torques*
		No change		Streptococcaceae		
Zuo *et al*., 2020	SARS-CoV-2	Increase	Actinobacteria Firmicutes Verrucomicrobia	Erysipelotrichaceae	*Coprobacillus* *Coprococcus* *Enterobacter* *Parabacteroides*	*Actinomyces odontolyticus* *Actinomyces viscosus* *Alistipes indistinctus* *Bacteroides nordii* *Clostridium hathewayi* *Clostridium ramosum* *Enterobacter cloacae*
		Decrease	Bacteroidetes	Lachnospiraceae	*Alloiococcus* *Dorea* *Faecalibacterium* *Roseburia*	*Alistipes onderdonkii* *Anaerovorax odorimutans* *Bacteroides ovatus* *Dorea longicatena* *Eubacterium ventriosum* *Faecalibacterium prausnitzii*
Zuo *et al*., 2020	CAP	Decrease			*Coprobacillus*	*Clostridium ramosum* *Erysipelotoclostrium ramosum* *Eubacterium ventriosum* *Enterococcus faecium* *Lachnospiraceae sp*.
Ren *et al*., 2020	CAP	Increase	Actinobacteria Proteobacteria	Actinomycetaceae Bifidobacteriaceae Enterobacteriaceae Enterococcaceae Leuconostocaceae Moraxellaceae Prevotellaceae Streptococcaceae	*Acetivibrio* *Acinetobacter* *Actinomyces* *Bacillus* *Bifidobacterium* *Enterococcus* *Escherichia* *Lactococcus* *Psychrobacter* *Scardovia* *Streptococcus* *Shigella*	*Actinomyces viscosus* *Subdoligranulum sp*.
		Decrease	Bacteroidetes Firmicutes Verrucomicrobia	Christensenellaceae Lachnospiraceae Ruminococcaceae Bacteroidaceae	*Bacteroides* *Clostridium* *Dorea* *Lachnospira* *Faecalibacterium* *Ruminococcus*	*Anaerostipes hadras*
Li L., *et al*., 2019	rRTIs	Increase	Bacteroidetes Proteobacteria	Streptococcaceae	*Bacteroides* *Enterococcus* *Escherichia* *Parabacteroides* *Shigella*	*Lachnoclostridium sp*. *Ruminococcus gnavus*
		Decrease	Actinobacteria Firmicutes Tenericutes Verrucomicrobia	Ruminococcaceae	*Actinomyces* *Blautia* *Butyricoccus* *Clostridium* *Corynebaterium* *Faecalibacterium* *Klebsiella* *Lactobacillus* *Prevotella* *Ruminococcus* *Veillonella* *Verrucomicrobiae*	*Erysipelotoclostrium ramosum* *Eubacterium rectale* *Subdoligranulum sp*.
Hu *et al*., 2019	TB	Increase	Actinobacteria Bacteroidetes Fusobacteria		*Actinomyces* *Bacteroides* *Bifidobacterium* *Blautia* *Oscillibacter* *Parabacteroides* *Paraprevotella* *Phascolarctobacterium* *Prevotella* *Scardovia* *Veillonella* *Verrucomicrobiae*	*Parascardovia sp*.
		Decrease	Firmicutes Proteobacteria Verrucomicrobia		*Alistipes* *Atopobium* *Collinsella* *Eubacterium* *Gardnerella* *Klebsiella* *Megamonas* *Roseburia* *Ruminococcus*	*Subdoligranulum sp*.
		No change		Coriobacteriaceae	*Adlercreutzia* *Agathobacter* *Clostridium* *Enterobacter* *Rothia*	
Li W., *et al*., 2019	TB	Increase	Bacteroidetes Proteobacteria	Enterococcaceae Prevotellaceae	*Enterococcus* *Prevotella*	
		Decrease	Actinobacteria Firmicutes	Bifidobacteriaceae Lachnospiraceae Rikenellaceae Ruminococcaceae	*Bacteroides* *Bifidobacterium* *Dorea* *Faecalibacterium* *Ruminococcus*	*Faecalibacterium prausnitzii*
Luo *et al*., 2017	New TB	Increase	Firmicutes Proteobacteria Verrucomicrobia		*Akkermansia* *Faecalibacterium* *Fusobacterium* *Parabacteroides* *Streptococcus*	
		Decrease	Bacteroidetes		*Prevotella*	
		No change			*Bacteroides* *Succinivibrio* *Veillonella* *Verrucomicrobiae*	
Luo *et al*., 2017	Recurrent TB	Increase	Actinobacteria Proteobacteria		*Akkermansia* *Streptococcus* *Veillonella* *Verrucomicrobiae*	
		Decrease	Bacteroidetes Firmicutes		*Faecalibacterium* *Parabacteroides* *Prevotella* *Succinivibrio*	
		No change			*Bacteroides* *Fusobacterium*	
Qin *et al*., 2015	Influenza H7N9	Increase	Firmicutes Proteobacteria		*Clostridium* *Enterococcus* *Escherichia* *Lactobacillus* *Parabacteroides* *Prevotella* *Shigella* *Streptococcus* *Veillonella*	*Enterococcus faecium*
		Decrease	Bacteroidetes		*Bacteroides* *Blautia* *Eubacterium* *Roseburia* *Ruminococcus*	
		No change	Actinobacteria		*Coprococcus* *Dorea* *Faecalibacterium*	

Taxa abundance variation was reported as increased (green), decreased (yellow), or no change (colourless). Abbreviations: Respiratory Tract Infection (RTI), Severe Acute Respiratory Syndrome Coronavirus 2 (SARS-CoV-2), Tuberculosis (TB), Community-Acquired Pneumonia (CAP), Recurrent-RTI (rRTI), Influenza A virus subtype H1N1, Influenza A virus subtype H7N9.

**Fig 2:**
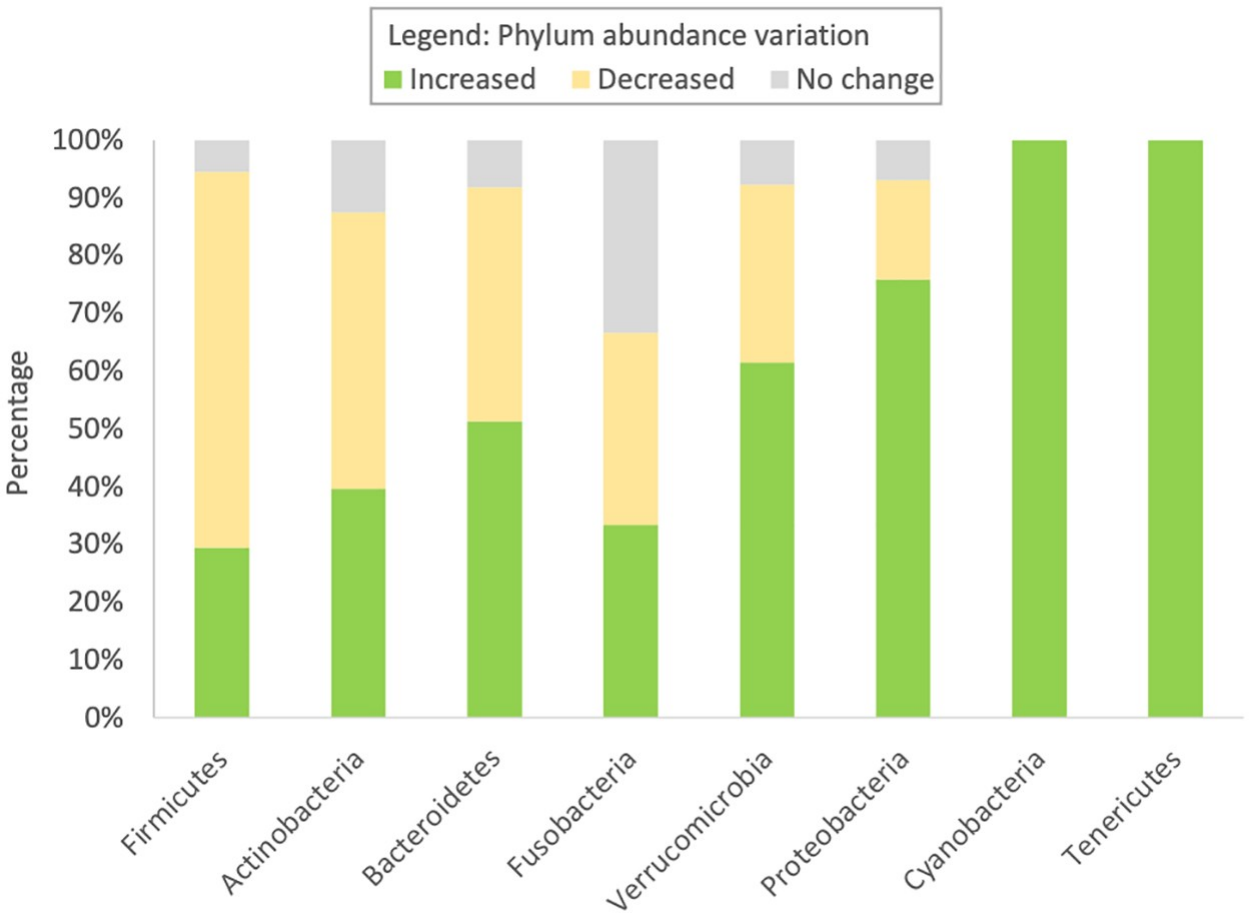
Percentage of gut microbiome phyla in patients with a respiratory tract infection compared to healthy participants. See supplementary information for a full breakdown of reported relative abundances (S1 Appendix and S3 Table).

Collectively, 14 microbiome data sets were considered for microbiome extraction and further analysis. We found a significant difference between the total reported number of gut bacteria in patients with an RTI reported as decreased (52%) compared to increased (41%) compared to healthy controls (Fisher’s exact probability, *p* = 0.0086, X^2^ = 5.67, α = 0.05, 95% CI, 0.0315–1), as seen in S3 Table. All reported bacteria were allocated into their corresponding phylum. The relative abundance of each phylum in RTI patients and healthy controls were compared. Most studies reported a ‘decrease’ in the level of Firmicutes in RTI patients compared to healthy controls (Fig 2).

The alpha diversity measures Shannon Diversity Index (DI), Chao1 and richness as measured by number of OTUs were tabulated (S4 Table). Heterogeneity on ten data sets (extracted from eight studies) using restricted maximum-likelihood (REML) in a random effects model using Shannon DI showed high heterogeneity between studies (I^2^, 94.42). Despite this the alpha diversity of gut microbiota in RTI patients was significantly lower 1.45 units (95% CI, 0.15–2.50) than healthy controls (*p* < 0.001).

**Fig 3:**
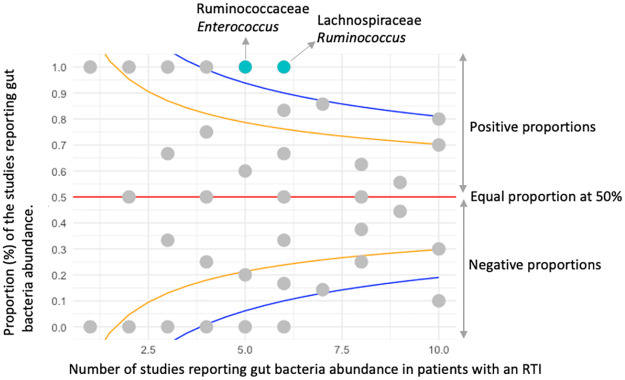
A funnel plot shows the number of studies reporting a change in gut bacteria abundance in patients with an RTI compared to healthy participants. The specified score2 (alpha2) confidence limits (Cl) are represented at, 50% (Cl) red line, 80% (Cl) orange line and 95% (Cl) blue line. Positive proportions are represented above the average line, with turquoise dots indicating studies reporting both increased and decreased gut bacteria (>95%, Cl). Whereas grey dots indicate studies reporting increased and decreased gut bacteria (<80%, Cl). There were 5/5 reports of an ‘increase’ in *Enterococcus*, 5/5 reports of a ‘decrease’ in Ruminococcaceae and 6/6 reports of a ‘decrease’ in both Lachnospiraceae and *Ruminococcus* in patients with an RTI compared to healthy participants. ● Abundance variation of gut bacteria in positive proportions at score2 of ≥ 95% confidence limits ● Abundance variation of gut bacteria of ≤ 80% confidence limits.

The proportion of studies that reported a specific gut bacterium as ‘increased’ or ‘decreased’ in RTI patients, out of the total number of studies is depicted in Fig 3. In RTI patients, *Enterococcus* was consistently reported as increased whereas those most reported as decreased were Ruminococcaceae, Lachnospiraceae and genus *Ruminococcus*.

A total of 11 studies and nested studies reported gut microbiota modifications in patients with the following respiratory pathogens, Severe Acute Respiratory Syndrome Coronavirus 2 (SARS-CoV-2) infection (n = 5), influenza infection (n = 2), *Mycobacterium tuberculosis* (TB) (n = 3), Community-Acquired Pneumonia CAP (n = 2) and recurrent RTIs (RRTI) (n = 1). Nested studies reported gut microbiota modifications from two different RTI-causing pathogens, SARS-CoV-2 and influenza (subtype H1N1) [46], SARS-CoV-2 and CAP [30], recurrent TB and new TB [47]. All reported gut bacteria diversity in the context of the specific RTI pathogen are described below.

### Covid-19

In 24 patients with laboratory confirmed SARS-CoV-2 compared to healthy controls the gut microbiome diversity was significantly reduced [48]. Ruminococcaceae and *Lachnospiraceae* including genus such as *Bifidobacterium*, *Blautia* and *Faecalibacterium* were depleted but both *Enterococcus* and *Streptococcus* had increased abundance. A larger study with 100 SARS-CoV-2 positive participants reported depletion of key taxa including *Faecalibacterium prausnitzii*, *Eubacterium rectale*, *Bifidobacteria* sp., and *Actinobacteria* whereas an increase was observed in *Akkermansia muciniphila*, *Bacteroides* sp., and *Ruminococcus* sp [49]. A earlier study with 30 Covid-19 patients reported a decrease of Actinobacteria and Firmicutes, including the genera *Bifidobacterium*, *Collinsella*, *Dorea*, *Faecalibacterium* and *Ruminococcus*, and species *Clostridium stricto* 1, *Eubacterium hallii*, *Ruminococcus torques* and whereas the abundance of *Streptococcus*, *Rothia*, *Veillonella*, and *Actinomyces* were increased [46]. Zuo *et al*., compared 15 Covid-19 patients with healthy controls and identified significant depletion of Bacteroidetes and genera *Dorea*, *Faecalibacterium* and *Roseburia* and an increase in Firmicutes, *Coprobacillus sp*., *Clostridium ramosum*, *Clostridium hathewayi*, *Actinomyces viscosus and Bacteroides nordii* [30]. A study on predominantly African American SARS-CoV-2 infected patients (n = 50) showed *Corynebacterium*, *Campylobacter* and *Finegoldia* were most significantly enriched while *Klebsiella*, *Agathobacter* and *Fusicatenibacter* were the top three genera significantly enriched in uninfected control patients (n = 34) [23].

### Community-Acquired Pneumonia

Zuo and colleagues also recruited 6 adult patients with CAP and compared the gut microbiome to healthy controls [30]. They found depletion of *Coprobacillus*, *Clostridium ramosum*, *Eubacterium ventriosum*, *Enterococcus faecium* and *Lachnospiraceae* sp. In contrast, another study of 11 children (4–5 years) with CAP, their gut microbiomes were depleted of phyla Bacteroidetes and Firmicutes plus genus *Dorea*, *Lachnospira*, *Faecalibacterium*, *Ruminococcus* and *Anaerostipes hadras* whereas enrichment was observed for phyla Actinobacteria and Proteobacteria, genera, *Streptococcus*, *Actinomyces*, *Enterococcus*, *Escherichia* and *Actinomyces viscosus*, *Subdoligranulum* sp. [50].

### Recurrent RTIs

In 26 children with a rRTI compared to healthy controls the gut community was depleted of phyla Actinobacteria, Firmicutes, Tenericutes and Verrucomicrobia and genera including *Faecalibacterium* and *Ruminococcus* yet enriched in phyla Bacteroidetes, Proteobacteria and genera *Escherichia* and *Enterococcus* [51].

### Tuberculosis

The gut microbiome of 46 adult patients with TB was compared to 31 healthy controls and found to depleted of phyla Firmicutes, Proteobacteria and Verrucomicrobia and nine genera, including *Collinsella*, *Roseburia*, *Ruminococcus* though increases were observed in phyla Actinobacteria, Bacteroidetes, Fusobacteria, genus including *Actinomyces*, *Bifidobacterium*, *Blautia*, *Parabacteroides* and species *Parascardovia* sp., [52]. In gut microbiota from patients with both new and recurrent TB (19 and 18, respectively) there was a decrease in Bacteroidetes, genus *Prevotella* and *Lachnospira* and enrichment in Actinobacteria and Proteobacteria [47]. In comparison another study recruited 18 children with TB and showed gut bacteria depleted in phyla Actinobacteria and Firmicutes, genera including Bacteroides, *Bifidobacterium*, *Dorea*, *Faecalibacterium*, *Ruminococcus* and *F*. *prausnitzii* yet enriched in Bacteroidetes, Proteobacteria, *Enterococcus* and *Prevotella* [53].

### Influenza

A study with 26 influenza subtype H7N9 confirmed patients compared to healthy controls showed a decrease in phyla Bacteroidetes and genus including *Bacteroides*, *Blautia*, *Roseburia* and *Ruminococcus* but enrichment of Firmicutes and Proteobacteria also genera *Escherichia*, *Clostridium* and *Enterococcus faecium* [31]. Another study with 24 influenza subtype H1N1 confirmed patients compared to healthy controls showed depletion of phyla Actinobacteria and Firmicutes, genera including *Dorea*, *Faecalibacterium*, *Ruminococcus*, *Streptococcus*, *Rothia* and species *Ruminococcus torques* whereas enrichment was observed for both Actinomycetaceae and Micrococcaceae [46].

## Discussion

This systematic review examined 11 studies that reported gut microbiome modifications in patients with a proven or suspected RTI, compared to healthy, matched controls. Collectively, reported gut microbiome modifications in patients with an RTI compared to healthy controls were consistently reported as decreased in diversity with a depletion of Firmicutes, Lachnospiraceae, Ruminococcaceae and *Ruminococcus* and enrichment of *Enterococcus*.

A key strength of this systematic review is that a comprehensive search ensured all relevant literature was identified including thorough unbiased analysis of reported microbiome taxa. While other systematic reviews have examined gut microbiome changes in the context of non-infectious respiratory diseases e.g., asthma and chronic obstructive pulmonary disease (COPD) or *in vitro* animal models, this novel systematic review was designed to evaluate reported gut microbiome modifications in studies that recruited patients with proven or suspected viral or bacterial respiratory infections including SARS-CoV-2, influenza, TB and CAP.

In patients with an RTI compared to controls we found a depletion of the family Ruminococcaceae which includes the genus *Ruminococcus* and *Faecalibacterium*. These bacteria produce SCFAs through the fermentation of dietary fibre and resistant starch. SCFAs play a key role in neuro-immunoendocrine regulation and are an important fuel for intestinal epithelial cells strengthening the gut barrier function [54]. In addition, SCFAs may signal through cell surface G-protein coupled receptors (GPCRs), like GPR41, to activate signalling cascades that control immune functions [55]. We found a decreased abundance of *R*. *torques* in a nested cohort study including RTIs of patients infected with SARS-CoV-2 or influenza (H1N1) [46] although one study reported an increased abundance in SARS-CoV-2 patients compared to healthy controls [49]. *R*. *torques* and *R*. *gnavus* have intramolecular trans-sialidase enzymes that allows for growth in the gastrointestinal mucus layer [56].

A notable SCFA-producing gut commensal *F*. *prausnitzii* was reported as having a decreased abundance compared to controls in cohorts of TB infected children and adults infected with SARS-CoV-2 [30, 49, 53]. *F*. *prausnitzii* is thought to have an anti-inflammatory effect, protecting against a range of gastrointestinal illnesses, including Crohn’s disease [55, 57]. Depletion of *F*. *prausnitzii* has been suggested to have a role in respiratory and lung diseases including asthma and cystic fibrosis [58, 59]. More recently, a study of Covid-19 recovered patients with ongoing symptoms reported that chest tightness post-activity was negatively correlated with the relative abundance of *F*. *prausnitzii* [60].

Lachnospiraceae were reported as depleted in patients with an RTI and are a common key component of the gut microbiota that ferment diverse plant polysaccharides to produce SCFAs and alcohols [61]. These beneficial butyrate-producers play a role in maintenance of a healthy gut microbiome suggesting they are essential for ongoing gut development and pathogen protection [62]. The genera *Dorea* and *Roseburia* are known SCFAs producers and have been reported as being depleted in respiratory disease including COPD [63]. Several of the reviewed studies reported a decrease in abundance of species *Dorea longicatena* in patients with an RTI compared to controls [30, 46, 49, 50, 53]. D. *longicatena* has been reported as a biomarker for obesity [64], yet it’s role in RTI is unknown.

Evidence suggests RTI-induced gut microbiome modifications results in depletion of protective gut commensals known to produce SCFAs. However, an *ex vivo* study found that addition of just SCFA molecules to SARS-CoV-2 infected human intestinal biopsies and intestinal epithelial cells did not affect viral load [65]. It is clear further cohort studies paired with *ex vivo* and *in vitro* research are required to fully understand the effect of microbial metabolites on antiviral and antibacterial activity including associated host interactions [66].

Conversely, it has been found that in RTI patients common opportunistic pathobionts proliferate in response to more nutrients and space caused by disease or infection when the host’s resistance is low [67]. In this review, we identified *Enterococcus* was consistently reported as enhanced in the gut microbiome for a range of patients infected with SARS-CoV-2, TB and CAP [31, 48, 50, 51, 53]. Although, interestingly in adults infected with CAP species *E*. *faecium* was reported as decreased abundance compared to healthy controls [30]. Not all *Enterococcus* species are pathobionts, but *Enterococcal* blooms have been reported in gastric and colorectal cancers [68]. *Enterococci* are clinically important as many strains have a high level of natural antibiotic resistance [29]. Interestingly, COVID-19-positive patients had a higher rate of vancomycin-resistant *E*. *faecium* infection compared to those who tested negative [69], suggesting that during- and post-RTI the risk of antimicrobial resistance may be increased. Specific microbes causing superinfection in COVID-19 patients usually require further treatment and health professionals have warranted coverage of antibiotics such as vancomycin, to treat atypical pneumonia and staphylococci infections [70].

The gut microbiome is intrinsically dynamic and longitudinal studies are best for discovering time-associated changes in microbial communities. Three studies discovered persistent gut microbiome modifications post-RTI symptoms [30, 49, 53]. One study was a limited pilot cohort of 7 patients infected with SARS-Cov-2 that demonstrated persist microbial dysbiosis past viral clearance, regardless of antibiotic treatment [30]. A recent paper describes dynamic gut microbiome changes for 90 days post onset of Covid-19 symptoms [21]. This paper was excluded in this review due to the lack of a reported healthy control cohort. However, comparisons were made between patients with persistently low and high viral loads, revealing that high viral loads were associated with a higher abundance of opportunistic pathogens *Collinsella aerofaciens*, *C*. *tanakaei*, *Morganella morganii* and *S*. *infantis*. Low viral load was associated with an enhanced abundance of SCFA producers *Parabacteroides merdae*, *Bacteroides stercoris*, *Lachnspiraceae bacterium* 1_1_57FAA and *Alistipes onderdonkii*. It is of note that the gut microbiome of SARS-CoV-2 infected patients show a depletion of SCFA producers and enhanced opportunistic pathogens. In a recent study by Chen *et al*., (2021) stool samples were collected from SARS-CoV-2 infected patients during acute phase, convalescence, and 6 months after hospital discharge. Gut microbiome dysbiosis was over a longer period than might be expected based on time to recovery [71]. These studies demonstrate RTIs are associated with long-term changes to the gut microbiota and possible semi-permanent loss of species. These studies are valuable, yet it is not clear whether RTI induced gut microbiota dysbiosis can cause long-term clinical complications and increase the risk of later-life chronic health issues.

We are aware of several limitations for this review. There are considerably fewer human studies compared to animal models, which investigate the effects of a RTI on the gut microbiome [17]. Consequently, with limited studies we have provided a narrative description of gut microbe modifications reported from patients with an RTI infection compared to healthy controls. It was not possible to build a substantial understanding of gut microbe modifications in patients with a specific RTI pathogen due to the small number of studies for each respiratory pathogen. Therefore, we focused on gut microbe modifications that were observed consistently, regardless of the causative respiratory pathogen.

Antibiotics and anti-viral medications are known to cause gut microbiome modifications, thus studies that reported patients with an RTI taking medications at the time of specimen collection were excluded from analysis [20, 23–25]. Unsurprisingly, the excluded studies reported significant differences between the diversity and abundance of the gut microbiome of patients with an RTI using medications compared to healthy controls. Cao *et al*., (2021) describe a feasibility study of 13 SAR-CoV-2 infected patients taking a cocktail of antibiotics such as moxifloxacin, piperacillin, levofloxacine and or antivirals, demonstrated considerable changes to the gut microbiome and a decrease in abundance of several taxa including *Roseburia sp*., *F*. *prausnitzii* and *Dorea formicigenerans*. High heterogeneity in our study could be due to undisclosed and not reported antibiotic usage as a recent review demonstrated antibiotic treatment-associated gut microbiota modifications and considerable heterogeneity between studies [29]. Subtle cohort changes might include age of participant, demographic variation, ethnicity, body mass index, and diet. It has been shown that studies describing the effect of dietary fibre on gut microbiota and metabolic regulation demonstrated high heterogeneity [72]. Further inconsistencies were found between microbiome methodologies, bioinformatic tools and 16S rRNA gene primer design. Unless confounder effects are controlled and reported in the results, published cohort studies should be interpreted with caution.

A range of bacterial gDNA extraction methods and two different microbiome profiling methods (16S rRNA profiling and metagenomic sequencing) were used in the reviewed studies. These factors can influence the resulting microbial profiles. The 16S-targeted approach is useful for broad profiling of bacterial microbiomes to the genus level whereas metagenomics can provide a deeper level analysis. There is a higher chance of bias in the 16S method as the 16S primers used can bias towards amplification from certain taxa, compared to the untargeted libraries used in metagenomics [73]. We found several bioinformatic tools and microbe identification databases were used in all included studies. Microbiome analysis method optimisation is an ongoing issue in the field of ‘omic’ research [74].

This is a rapidly developing field, and our review has scratched the surface highlighting the lack of human cohort studies that definitively track gut microbiome changes during an RTI infection. The small number of studies that satisfied our inclusion criteria has emphasised the requirement for more cohort studies designed to identify gut microbiome modifications arising during RTIs, so that we can better understand interplay between microbes, respiratory health and the use of interventions such as pre- and probiotics. Also, longitudinal epidemiological microbiome studies could be used as a key tool for the identification of healthy patients most vulnerable to RTIs. Other studies might include:

1. Longitudinal studies that include pre-infection and post-infection gut microbiota profiles. This would enable identification of RTI-associated changes and subsequent recovery within the same patient.
2. Community and urban environment studies designed to consider social and demographic factors, including age, ethnicity, diet and deprivation scores.
3. Studies that consider RTI symptom clinical severity stratification (this was addressed by one article [49]).
4. Pathogen-specific studies. Here, the ten studies included encompassed 4 different pathogens, including both bacteria and viruses. Despite this, we identified consistent gut microbiota profile changes suggesting that generic infection induced changes. However, it is likely that different pathogens have more specific effects on the microbiota, and thus more pathogen-specific studies minus confounding effects are required to define these.

## Conclusions

We have found that patients with an RTI compared to healthy controls have a reported decrease in gut bacteria diversity and abundance within the phylum Firmicutes and unbiased analysis indicates a loss of Lachnospiraceae, Ruminococcaceae and *Ruminococcus* and an increase in *Enterococcus*. High heterogeneity between studies may be explained by variations in by microbiome methods and confounder effects. A full comprehensive report of gut microbiome modifications due to respiratory infection was limited due to lack of suitable cohort studies therefore further human studies in this area are urgently required.

## Supporting information

S1 Checklist PRISMA checklist. (DOCX)

S1 Appendix Summary of studies based on eligibility criteria and author best judgement (non-exhaustive list). Abbreviations: Community-acquired pneumonia (CAP), Severe acute respiratory syndrome coronavirus 2 (SARS-CoV-2), Mycobacterium tuberculosis (TB), recurrent-respiratory tract infection (RRTI).
(DOCX)

S1 Fig Study quality and risk of bias based on CASP checklist. Cohort studies (top) and case-controlled studies (bottom).
(TIF)

S1 Table Literature search strategy including (a) Medline, Embase and (b) Clinical trial databases. This search was performed twice in 6 months to cover the increase in COVID-19 publications and (b) clinical trial databases included WHO International Clinical Trials, UK Government Clinical Trials Platform and The Cochrane Central Register of Controlled Trials (CENTRAL).
(DOCX)

S2 Table Population, Intervention, Comparator, Outcome and Study (PICOS) design criteria for inclusion and exclusion. *Studies published from 2015 were included because microbiome analysis became established and methods consistent.
(DOCX)

S3 Table Change in relative abundance of gut bacteria in patients with a respiratory tract infection (RTI) compared to healthy participants. Reported increase and decrease abundance columns are shaded in green and yellow respectively and the taxa and total report columns are shaded grey. ^a^Total number of reports of each taxa in patients with an RTI compared to healthy controls from 11 articles (3 nested studies). ^b^Phylum allocation was applied to all reported taxa at the level of family, genus or species. ^c^ The total reported number of gut bacteria decreased compared to those increased was significantly different.
(DOCX)

S4 Table Gut microbiome alpha diversity in RTI patients compared to healthy controls. ^a^Alpha diversity in RTI patients gut microbiome was significantly different (p < 0.001) at 1.45 units (95% CI, 0.15–2.5) lower than in healthy controls. Shannon values were tested for heterogeneity using REML (restricted maximum-likelihood) in a random effects model (I^2^, 94.42, Cochranes Q = 50.70). ^b^Gut microbiome alpha diversity in patients with an RTI compared to healthy controls was not reported as significant in the study. Abbreviations: ND = not determined, operational taxonomic units (OTUs), Shannon diversity index (DI), Respiratory Tract Infection (RTI), Severe Acute Respiratory Syndrome Coronavirus 2 (SARS-CoV-2), *Tuberculosis* (TB), new *Tuberculosis* (nTB), recurrent *Tuberculosis* (rTB), Community-Acquired Pneumonia (CAP), Influenza A virus subtype (H1N1), Influenza A virus subtype (H7N9).
(DOCX)
