# Supplementary material for: Respiratory tract infections and gut microbiome modifications: A systematic review
Source: PLoS One. 2022 Jan 13;17(1):e0262057. doi: 10.1371/journal.pone.0262057 (PMC8757905; doi:10.1371/journal.pone.0262057)
Supplement: S1 Appendix — Abbreviations: Community-acquired pneumonia (CAP), Severe acute respiratory syndrome coronavirus 2 (SARS-CoV-2), Mycobacterium tuberculosis (TB), recurrent-respiratory tract infection (RRTI). (DOCX) [file pone.0262057.s002.docx]

| **No.** | **Author, (publication year)**  **[DOI]** | **Title** | **Country** | **Respiratory pathogen** | **Included in review (Y/N)** | **Reason for exclusion** |
| --- | --- | --- | --- | --- | --- | --- |
| 1 | Ren *et al*., (2021)  [10.1136/gutjnl-2020-323826] | Alterations in the human oral and gut microbiomes and lipidomics in COVID-19. | China | SARS-CoV-2 | Y | n/a |
| 2 | Yeoh *et al*., (2021)  [10.1136/gutjnl-2020-323020] | Gut microbiota composition reflects disease severity and dysfunctional immune responses in patients with COVID-19 | China | SARS-CoV-2 | Y | n/a |
| 3 | Gu *et al*., (2020)  [10.1093/cid/ciaa709] | Alterations of the Gut Microbiota in Patients with COVID-19 or H1N1 Influenza. | China | SARS-CoV-2  &  Influenza (H1N1) | Y | n/a |
| 4 | Ren *et al.,* (2020)  [10.1016/j.micpath.2020.104062] | The distribution characteristics of intestinal microbiota in children with community-acquired pneumonia under five Years of age. | Inner Mongolia | CAP | Y | The gut microbiome data of children 4-5 years old were included. Those children who were <4 years old gut microbiome data was excluded. |
| 5 | Zuo *et al*., (2020)  [10.1053/j.gastro.2020.05.048] | Alterations in Gut Microbiota of Patients With COVID-19 During Time of Hospitalization | China | SARS-CoV-2  &  CAP | Y | n/a |
| 6 | Hu *et al*., (2019)  [10.3389/fcimb.2019.00090] | The Gut Microbiome Signatures Discriminate Healthy From Pulmonary Tuberculosis Patients. | China | TB | Y | n/a |
| 7 | Li *et al*., (2019)  [10.1016/j.micpath.2019.103709] | Intestinal microbiota dysbiosis in children with recurrent respiratory tract infections. | China | RRTI | Y | n/a |
| 8 | Li *et al*., (2019)  [10.1186/s12887-019-1782-2] | Characterization of gut microbiota in children with pulmonary tuberculosis. | China | TB | Y | n/a |
| 9 | Luo *et al*., (2017)  [10.3389/fphys.2017.00822] | Alternation of gut microbiota in patients with pulmonary tuberculosis. | China | TB | Y | n/a |
| 10 | Qin *et al*., (2015)  [10.1038/srep14771] | Influence of H7N9 virus infection and associated treatment on human gut microbiota. | China | Influenza (H7N1) | Y | n/a |
| 11 | Cao *et al*., (2021)  [10.1080/19490976.2021.1887722] | Integrated gut virome and bacteriome dynamics in COVID-19 patients. | China | SARS-CoV-2 | N | All patients were taking antiviral drugs, 5 patients were taking antibiotics and the remaining 8 patients were not taking antibiotics. They found antibiotics resulted in a lower alpha diversity compared to controls. However, didn't consider the antivirals drugs as a possible confounder.  Patients were stratified by COVID-19 severity symptoms. A mouse model was included. |
| 12 | Zuo *et al*., (2020)  [10.1136/gutjnl-2020-322294] | Depicting SARS-CoV-2 faecal viral activity in association with gut microbiota composition in patients with COVID-19. | China | SARS-CoV-2 | N | A longitudinal analysis of COVID-19 patients. Metagenome analysis of the gut microbiome was compared between different levels of viral infection and a healthy control cohort was not used for comparison. |
| 13 | Marotz *et al*., (2021)  [10.1186/s40168-021-01083-0] | SARS-CoV-2 detection status associates with bacterial community composition in patients and the hospital environment. | USA | SARS-CoV-2 | N | An environmental study and human study combined. Most patients received one antibiotic while in hospital with a median stay of 9 days. Only one bacterium was associated with SARS-CoV-2 positive patients (*Rothia dentocariosa*). |
| 14 | Newsome *et al*., (2021)  [10.1080/19490976.2021.1926840] | The gut microbiome of COVID-19 recovered patients returns to uninfected status in a minority-dominated United States cohort. | USA | SARS-CoV-2 | Y | n/a |
| 15 | Mazzarelli *et al*., (2021)  [10.1371/journal.pone.0247041] | 16S rRNA gene sequencing of rectal swab in patients affected by COVID-19. | Italy | SARS-CoV-2 | N | Antibiotics usage by 48% of cohort at least one / two days before the rectal swab was collected. Antibiotic usage was not adjusted in microbiome analysis. |
| 16 | Xu *et al*., (2021)  [10.1038/s42003-021-01796-w] | Temporal association between human upper respiratory and gut bacterial microbiomes during the course of COVID-19 in adults. | China | SARS-CoV-2 | N | Antibiotics usage by 85.7% of patients. Throat and anal swabs collected. Antibiotic usage was not adjusted in microbiome analysis. |
