# Supplementary figures and images for: Respiratory tract infections and gut microbiome modifications: A systematic review
Source: PLoS One. 2022 Jan 13;17(1):e0262057. doi: 10.1371/journal.pone.0262057 (PMC8757905; doi:10.1371/journal.pone.0262057)

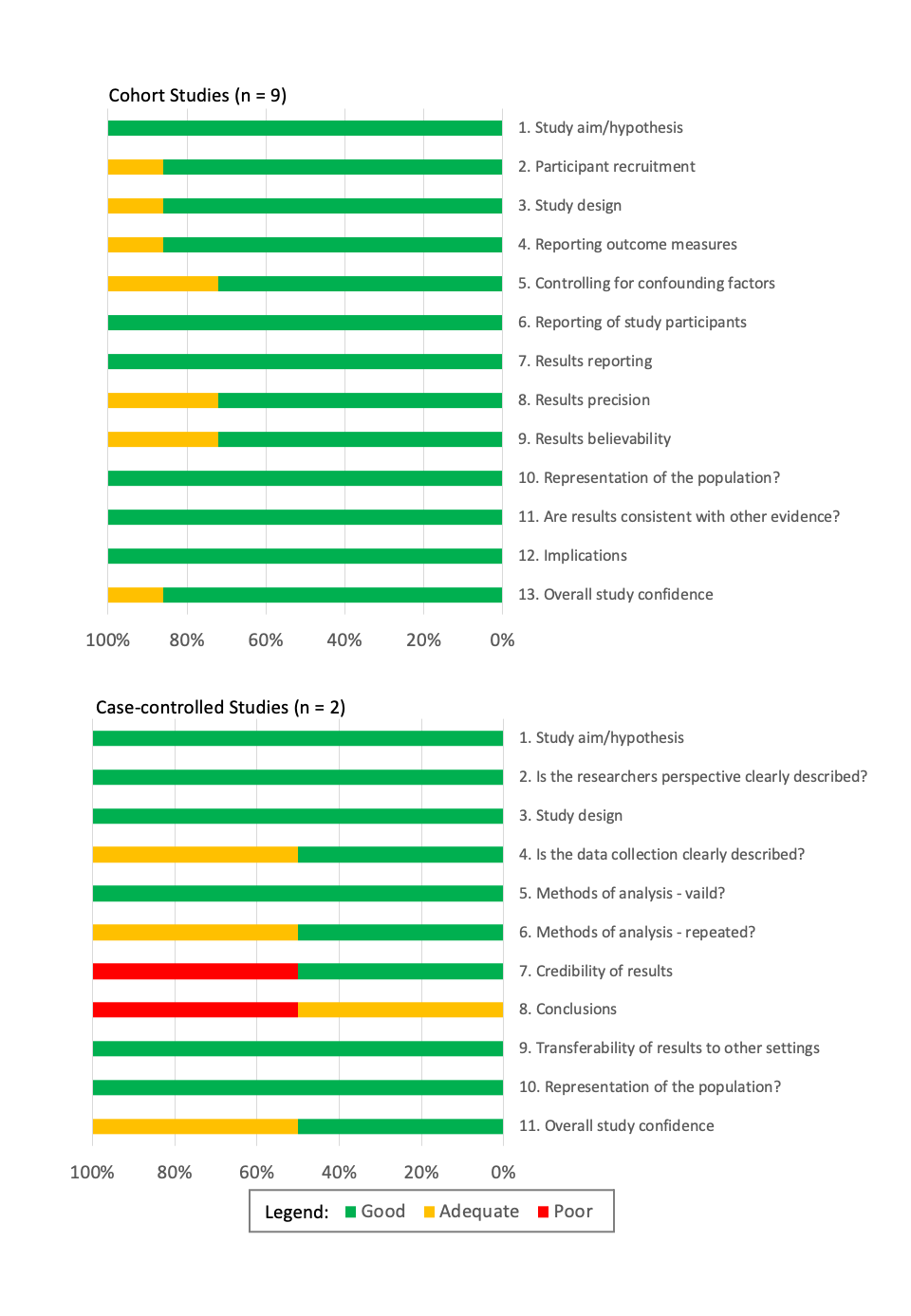

Supplement: S1 Fig — Cohort studies (top) and case-controlled studies (bottom). (TIF) [file pone.0262057.s003.tif]
