# Supplementary material for: Respiratory tract infections and gut microbiome modifications: A systematic review
Source: PLoS One. 2022 Jan 13;17(1):e0262057. doi: 10.1371/journal.pone.0262057 (PMC8757905; doi:10.1371/journal.pone.0262057)
Supplement: S1 Table — This search was performed twice in 6 months to cover the increase in COVID-19 publications and (b) clinical trial databases included WHO International Clinical Trials, UK Government Clinical Trials Platform and The Cochrane Central Register of Controlled Trials (CENTRAL). (DOCX) [file pone.0262057.s004.docx]

**(a)**

| # | Search Term | Articles retrieved |
| --- | --- | --- |
| 1 | microbiom$.mp. | 48,394 |
| 2 | (microbiota* or micro-biota* or microbiome* or microbe or bacteria* or biologic* or microbiologic* or micro-biology* or microbiology*).ti,ab,kw. | 1,713,206 |
| 3 | respiratory.mp. | 603,917 |
| 4 | exp Respiratory Tract Infections/ | 450,974 |
| 5 | (bronchi* or pneumonia or croup or throat or tonsillitis or pharyngitis or nasopharyngitis or laryngitis or tracheitis or sinusitis or rhino*).ti,ab,kw. | 345,261 |
| 6 | (RSV or influenza or rhinovirus or virus or covid* or corona or COVID* or SAR* or covid-19 or MER* or HCoVNL* or COVNL63).mp. | 1,578,561 |
| 7 | exp intestine/ | 398,244 |
| 8 | (gut or intestine or gastrointestinal or faec* or stool or fecal).mp. | 709,768 |
| 9 | 1 or 2 | 1,715,568 |
| 10 | 3 or 4 or 5 or 6 | 2,475,022 |
| 11 | 7 or 8 | 935,711 |
| 12 | 9 and 10 and 11 | 10,212 |
| 13 | limit 12 to English language | 9,305 |
| 14 | limit 13 to humans | 5,485 |
| 15 | limit 14 to yr="2015 - Current" | 2,414 |
| 16 | limit 15 to "review articles" | 819 |
| 17 | 15 not 16 | 1,595 |

**(b)**

| Number | Search Term |
| --- | --- |
| 1 | Respiratory tract infection |
| 2 | Microbiome |
| 3 | Respiratory tract infection + microbiome |
| 4 | Gut |
| 5 | Microbiome + gut |
| 6 | Respiratory tract infection + gut |
