# Supplementary material for: Respiratory tract infections and gut microbiome modifications: A systematic review
Source: PLoS One. 2022 Jan 13;17(1):e0262057. doi: 10.1371/journal.pone.0262057 (PMC8757905; doi:10.1371/journal.pone.0262057)
Supplement: S2 Table — *Studies published from 2015 were included because microbiome analysis became established and methods consistent. (DOCX) [file pone.0262057.s005.docx]

| **PICOS** | **Inclusion criteria** | **Exclusion criteria** |
| --- | --- | --- |
| Population | Humans only. Children > 3 years old, up to and including adults. Any country. | Babies, neonates, children < 3 years old. |
| Exposure | Individuals with a possible suspected, symptoms of, or clinically confirmed respiratory tract infection (RTI) combined with microbiome analysis of stool samples proxy gastrointestinal tract.  Eligible RTIs included but was not limited to: coronaviruses such as Middle East respiratory syndrome-related coronavirus, severe acute respiratory syndrome coronavirus (SARS-CoV-1) and human coronavirus NL63; influenza (any subtype), common cold, Whooping cough caused by *Bordetella pertussis*, respiratory syncytial virus (RSV); tuberculosis caused by *Mycobacterium tuberculosis* (TB); pneumonia caused by a bacterial or viral pathogen; bronchiectasis caused by pneumonia or tuberculosis; chronic cough caused by bacteria or viral pathogen. | Respiratory disease including chronic obstructive pulmonary disease, lung cancer, mesothelioma, sarcoidosis, asthma, cystic fibrosis, bronchiectasis, chronic/acute bronchitis, emphysema, pleural effusion, idiopathic pulmonary fibrosis, pulmonary oedema or interstitial lung disease. Environmental and occupational exposures causing lung disease, including smoking. Mechanical ventilation. Sleep apnoea. Autoimmune diseases. HIV infection or AIDS. Hantavirus pulmonary syndrome. Legionnaires’ disease. Allergies, aspergillosis, coccidioidomycosis, histoplasmosis.  Faecal microbial transplants. Interventions such as antibiotics, probiotics, diet, herbal. Or studies that compared age ranges and or body mass index. |
| Comparator | Participants who did not have a RTI and were identified as a healthy control (usually age and gender matched). |  |
| Outcomes | Gut microbiome diversity, taxonomic phylogeny and abundance. | No gut microbiome data, no taxonomic identification of bacteria. |
| Study design | Studies published from 2015* – March 2021.  Observational studies   - Cohort - Case-control - Cross-sectional - Longitudinal   Randomised or non-randomised trials encompassed by the above. | Studies before 2015. Systematic reviews, notes to journal, conference abstracts. Economic studies. Critical reviews, expert opinions without primary data. Qualitative or any studies without microbiome data. |
