# Supplementary material for: Respiratory tract infections and gut microbiome modifications: A systematic review
Source: PLoS One. 2022 Jan 13;17(1):e0262057. doi: 10.1371/journal.pone.0262057 (PMC8757905; doi:10.1371/journal.pone.0262057)
Supplement: S3 Table — Reported increase and decrease abundance columns are shaded in green and yellow respectively and the taxa and total report columns are shaded grey. aTotal number of reports of each taxa in patients with an RTI compared to healthy controls from 11 articles (3 nested studies). bPhylum allocation was applied to all reported taxa at the level of family, genus or species. c The total reported number of gut bacteria decreased compared to those increased was significantly different. (DOCX) [file pone.0262057.s006.docx]

| **Gut bacteria** | **Taxa** | **Phylum allocation^b^** | **Increased**  **abundance** | **Decreased**  **abundance** | **No change** | **Total reports^a^** |
| --- | --- | --- | --- | --- | --- | --- |
| Actinobacteria | Phylum | A | 3 | 5 | 1 | 8 |
| Bacteroidetes |  | B | 4 | 5 | 0 | 9 |
| Cyanobacteria |  | C | 1 | 0 | 0 | 1 |
| Firmicutes |  | F | 3 | 7 | 0 | 10 |
| Fusobacteria |  | Fu | 1 | 0 | 0 | 1 |
| Proteobacteria |  | P | 6 | 1 | 0 | 7 |
| Tenericutes |  | T | 1 | 0 | 0 | 1 |
| Verrucomicrobia |  | V | 3 | 3 | 0 | 6 |
| Actinomycetaceae | Family | A | 3 | 0 | 0 | 3 |
| Bacteroidaceae |  | B | 0 | 0 | 1 | 1 |
| Bifidobacteriaceae |  | A | 1 | 3 | 0 | 4 |
| Christensenellaceae |  | F | 0 | 1 | 0 | 1 |
| Coriobacteriaceae |  | A | 0 | 2 | 1 | 3 |
| Enterobacteriaceae |  | P | 1 | 0 | 0 | 1 |
| Enterococcaceae |  | F | 2 | 0 | 0 | 2 |
| Erysipelotrichaceae |  | F | 1 | 2 | 0 | 3 |
| Lachnospiraceae |  | F | 0 | 5 | 0 | 5 |
| Leuconostocaceae |  | F | 1 | 0 | 0 | 1 |
| Micrococcaceae |  | A | 2 | 0 | 0 | 2 |
| Moraxellaceae |  | P | 1 | 0 | 0 | 1 |
| Peptostreptococcaceae |  | F | 0 | 2 | 0 | 2 |
| Prevotellaceae |  | B | 2 | 0 | 0 | 2 |
| Rikenellaceae |  | B | 0 | 1 | 0 | 1 |
| Ruminococcaceae |  | F | 0 | 5 | 0 | 5 |
| Streptococcaceae |  | F | 3 | 0 | 1 | 4 |
| *Acetivibrio* | Genus | F | 1 | 0 | 0 | 1 |
| *Acinetobacter* |  | P | 1 | 0 | 0 | 1 |
| *Actinomyces* |  | A | 2 | 1 | 0 | 3 |
| *Adlercreutzia* |  | A | 0 | 0 | 1 | 1 |
| *Agathobacter* |  | F | 0 | 2 | 1 | 3 |
| *Akkermansia* |  | V | 2 | 0 | 1 | 3 |
| *Alistipes* |  | B | 0 | 1 | 0 | 1 |
| *Alloiococcus* |  | F | 0 | 1 | 0 | 1 |
| *Anaerostipes* |  | F | 0 | 2 | 0 | 2 |
| *Atopobium* |  | A | 0 | 1 | 0 | 1 |
| *Bacillus* |  | F | 1 | 0 | 0 | 1 |
| *Bacteroides* |  | B | 2 | 4 | 2 | 8 |
| *Bifidobacterium* |  | A | 2 | 4 | 0 | 6 |
| *Blautia* |  | F | 4 | 2 | 0 | 6 |
| *Butyricoccus* |  | F | 0 | 1 | 0 | 1 |
| *Campylobacter* |  | P | 0 | 1 | 0 | 1 |
| *Clostridium* |  | F | 1 | 2 | 1 | 4 |
| *Collinsella* |  | A | 0 | 3 | 0 | 3 |
| *Coprobacillus* |  | F | 1 | 1 | 0 | 2 |
| *Coprococcus* |  | F | 1 | 0 | 1 | 2 |
| *Corynebaterium* |  | A | 1 | 1 | 0 | 2 |
| *Dorea* |  | F | 0 | 5 | 1 | 6 |
| *Enterobacter* |  | P | 1 | 0 | 1 | 2 |
| *Enterococcus* |  | F | 5 | 0 | 0 | 5 |
| *Erysipelotoclostrium* |  | F | 0 | 2 | 0 | 2 |
| *Escherichia* |  | P | 3 | 0 | 0 | 3 |
| *Eubacterium* |  | F | 0 | 2 | 0 | 2 |
| *Faecalibacterium* |  | F | 1 | 8 | 1 | 10 |
| *Finegoldia* |  | F | 1 | 0 | 0 | 1 |
| *Fusobacterium* |  | Fu | 0 | 1 | 1 | 2 |
| *Fusicatenibacter* |  | F | 0 | 2 | 0 | 2 |
| *Gardnerella* |  | A | 0 | 1 | 0 | 1 |
| *Intestinibacter* |  | F | 0 | 2 | 0 | 2 |
| *Klebsiella* |  | P | 0 | 2 | 0 | 2 |
| *Lachnospira* |  | F | 0 | 1 | 0 | 1 |
| *Lachnospiraceae incertae sedis* |  | F | 0 | 1 | 0 | 1 |
| *Lactobacillus* |  | F | 1 | 1 | 0 | 2 |
| *Lactococcus* |  | F | 1 | 0 | 0 | 1 |
| *Megamonas* |  | F | 0 | 1 | 0 | 1 |
| *Oscillibacter* |  | F | 1 | 0 | 0 | 1 |
| *Parabacteroides* |  | B | 5 | 1 | 0 | 6 |
| *Paraprevotella* |  | B | 1 | 0 | 0 | 1 |
| *Phascolarctobacterium* |  | F | 1 | 0 | 0 | 1 |
| *Prevotella* |  | B | 3 | 3 | 0 | 6 |
| *Pseudobutyrivibrio* |  | F | 0 | 1 | 0 | 1 |
| *Psychrobacter* |  | B | 1 | 0 | 0 | 1 |
| *Roseburia* |  | F | 0 | 3 | 0 | 3 |
| *Romboutsia* |  | F | 0 | 2 | 0 | 2 |
| *Rothia* |  | A | 0 | 0 | 3 | 3 |
| *Ruminococcus* |  | F | 0 | 6 | 0 | 6 |
| *Scardovia* |  | A | 2 | 0 | 0 | 2 |
| *Shigella* |  | P | 3 | 0 | 0 | 3 |
| *Streptococcus* |  | F | 6 | 1 | 0 | 7 |
| *Succinivibrio* |  | P | 0 | 1 | 1 | 2 |
| *Veillonella* |  | F | 3 | 1 | 1 | 5 |
| *Verrucomicrobiae* |  | V | 2 | 1 | 1 | 4 |
| *Actinomyces odontolyticus* | Species | A | 1 | 0 | 0 | 1 |
| *Actinomyces viscosus* |  | A | 2 | 0 | 0 | 2 |
| *Akkermansia muciniphila* |  | V | 1 | 0 | 0 | 1 |
| *Alistipes indistinctus* |  | B | 1 | 0 | 0 | 1 |
| *Alistipes onderdonkii* |  | B | 0 | 1 | 0 | 1 |
| *Anaerostipes hadras* |  | F | 0 | 1 | 0 | 1 |
| *Anaerovorax odorimutans* |  | F | 0 | 1 | 0 | 1 |
| *Bacteroides dorei* |  | P | 1 | 0 | 0 | 1 |
| *Bacteroides caccce* |  | P | 1 | 0 | 0 | 1 |
| *Bacteroides nordii* |  | P | 1 | 0 | 0 | 1 |
| *Bacteroides ovatus* |  | P | 1 | 1 | 0 | 2 |
| *Bacteroides vulgatus* |  | P | 1 | 0 | 0 | 1 |
| *Bifidobacterium adolescentis* |  | A | 0 | 1 | 0 | 1 |
| *Bifidobacterium pseudocatenulatum* |  | A | 0 | 1 | 0 | 1 |
| *Clostridium hathewayi* |  | F | 1 | 0 | 0 | 1 |
| *Clostridium ramosum* |  | F | 1 | 1 | 0 | 2 |
| *Clostridium sensu stricto 1* |  | F | 0 | 2 | 0 | 2 |
| *Collinsella aerofaciens* |  | A | 0 | 1 | 0 | 1 |
| *Coprococcus comes* |  | F | 0 | 0 | 1 | 1 |
| *Dorea longicatena* |  | F | 0 | 2 | 0 | 2 |
| *Dorea formicigenerans* |  | F | 0 | 1 | 0 | 1 |
| *Enterobacter cloacae* |  | P | 1 | 0 | 0 | 1 |
| *Erysipelotoclostridium ramosum* |  | F | 0 | 2 | 0 | 2 |
| *Eubacterium hallii* |  | F | 0 | 2 | 0 | 2 |
| *Eubacterium rectale* |  | F | 0 | 2 | 0 | 2 |
| *Eubacterium ventriosum* |  | F | 0 | 2 | 0 | 2 |
| *Faecalibacterium prausnitzii* |  | F | 0 | 3 | 0 | 3 |
| *Lachnoclostridium sp.* |  | F | 1 | 0 | 0 | 1 |
| *Parascardovia sp.* |  | A | 1 | 0 | 0 | 1 |
| *Ruminococcus bromii* |  | F | 0 | 1 | 0 | 1 |
| *Ruminococcus gnavus* |  | F | 2 | 0 | 0 | 2 |
| *Ruminococcus obeum* |  | F | 0 | 1 | 0 | 1 |
| *Ruminococcus torques* |  | F | 1 | 2 | 0 | 3 |
| *Subdoligranulum sp.* |  | F | 2 | 4 | 0 | 4 |
| Total^c^ |  | 115 | 119 | 150 | 21 | 289 |
