# Supplementary material for: Respiratory tract infections and gut microbiome modifications: A systematic review
Source: PLoS One. 2022 Jan 13;17(1):e0262057. doi: 10.1371/journal.pone.0262057 (PMC8757905; doi:10.1371/journal.pone.0262057)
Supplement: S4 Table — aAlpha diversity in RTI patients gut microbiome was significantly different (p < 0.001) at 1.45 units (95% CI, 0.15–2.5) lower than in healthy controls. Shannon values were tested for heterogeneity using REML (restricted maximum-likelihood) in a random effects model (I2, 94.42, Cochranes Q = 50.70). bGut microbiome alpha diversity in patients with an RTI compared to healthy controls was not reported as significant in the study. Abbreviations: ND = not determined, operational taxonomic units (OTUs), Shannon diversity index (DI), Respiratory Tract Infection (RTI), Severe Acute Respiratory Syndrome Coronavirus 2 (SARS-CoV-2), Tuberculosis (TB), new Tuberculosis (nTB), recurrent Tuberculosis (rTB), Community-Acquired Pneumonia (CAP), Influenza A virus subtype (H1N1), Influenza A virus subtype (H7N9). (DOCX) [file pone.0262057.s007.docx]

| First author | Respiratory pathogen | Gut microbiome alpha diversity value (+ SE)^a^ | | | | | |
| --- | --- | --- | --- | --- | --- | --- | --- |
|  |  | Shannon DI | | OTUs | | Chao1 | |
|  |  | RTI | Healthy | RTI | Healthy | RTI | Healthy |
| Ren Z. | SARS-CoV-2 | 1.90 + 0.40 | 3.56 + 0.50 | ND | ND | ND | ND |
| Gu | SARS-CoV-2 | 3.10 | 3.80 | 911 | 922 | 285 | 390 |
| Ren X. | CAP | 3.12 ± 0.32 | 4.98 ± 0.16 | 250.5 ± 22.87 | 277.8 ± 16.04 | 297.7 | 333.9 |
| Hu | TB | 1.88 ± 0.53 | 2.05 ± 0.47 | 65 ± 15 | 90 ± 15 | ND | ND |
| Li W. | TB | 4.4 ± 0.87 | 5.4 ± 0.90 | 592 ± 259 | 716 ± 235 | 751 | 931 |
| Luo | nTB | 5.50^b^ | 5.54^b^ | 632 | 533 | 520 | 500 |
| Luo | rTB | 5.00^b^ | 5.54^b^ | 675 | 533 | 540 | 500 |
| Li L. | RRTIs | 2.19 ± 0.87 | 2.63 ± 0.78 | 280 | 311 | 104.79 ± 36.02 | 162.67 ± 51.99 |
| Gu | Influenza (H1N1) | 2.20 | 3.80 | 960 | 922 | 120 | 390 |
| Qin | Influenza (H7N9) | 2.10^b^ | 2.30^b^ | ND | ND | ND | ND |
